# Notes

**Published:** 1901-11

**Authors:** 


### NOTES.

New Milk Adulterant. A milk adulterant has been discovered by the dairy inspectors in Minnesota. It is called viscogen, and is composed of sugar, lime, and water. It has the effect of making milk appear richer than it is, as the lactic acid in the milk turns the lime to a thick, white substance that assimilates with the milk and improves its looks, while it does not injure the taste.

The Rio Chemical Company, formerly of St. Louis, Mo., have removed their establishment to 56 Thomas Street, New York City, in order that they may extend more readily the field for their products. The company issues a beautiful album, entitled "A Gallery of Pictures of Interest to Medical Men," which might well be in the possession of every veterinarian.

During the last few years tannoform (tannin-formaldehyde) has won great favor in its various applications. Reports from numerous sources indicate that it is rapid and certain in its effects in the diarrhœa of cattle and in infectious intestinal catarrhs of horses. Externally it is largely employed in purulent sores, recent wounds, eczemas, and as an antiseptic. A report has been made by M. H. Hayes, F.R.C.V.S., as follows: "I have used tannoform with 238 Government mounts, of which I was in veterinary charge on board H. M. T. No. 38, 'Idaho,' going to South Africa, and found it an excellent antiseptic. The fact of its being soluble in spirit is a great point in its favor."

Some further advantages in considering the external uses of tannoform are that it is odorless and twice as bulky as iodoform.

